# Damage evolution during fracture by correlative microscopy with hyperspectral electron microscopy and laboratory-based microtomography

**DOI:** 10.1126/sciadv.abj6738

**Published:** 2022-04-06

**Authors:** Peter M. Sarosi, Jevan Furmanski, William C. Reese, Donald L. Carpenter, Mikel A. Nittoli, Michael G. Myers, Nicole M. Callen, Thirumalai Neeraj

**Affiliations:** 1 Corporate Strategic Research, ExxonMobil Research and Engineering Company, Annandale, NJ 08801, USA.; 2 Research and Technology Department, ExxonMobil Upstream Integrated Solutions, Spring, TX 77389, USA.

## Abstract

Damage evolution during fracture of metals is a critical factor in determining the reliability and integrity of the infrastructure that the society relies upon. However, experimental techniques for directly observing these phenomena have remained challenged. We have addressed this gap by developing a correlative microscopy framework combining high-resolution hyperspectral electron microscopy with laboratory x-ray microtomography (XMT) and applied it to study fracture mechanisms in a steel inclusion system. We observed damage nucleation and growth to be inhomogeneous and anisotropic. Fracture resistance was observed to be controlled by inclusion distribution and the size scale of an inclusion-depleted zone. Furthermore, our studies demonstrate that laboratory XMT can characterize damage to the micrometer scale with a large field of view in dense metals like steel, offering a more accessible alternative to synchrotron-based tomography. The framework presented provides a means to broadly adopt correlative microscopy for studies of degradation phenomena and help accelerate discovery of new materials solutions.

## Teaser

Correlative microscopy using lab x-ray microtomography provides accessible characterization of damage phenomena in materials.

## INTRODUCTION

Metals and their alloys are most commonly used as engineering structural materials, as they offer an exceptional combination of strength and toughness (*1*, *2*). It is estimated that unexpected failures in infrastructure lead to billions of dollars in economic losses in the United States and on the order of trillions of dollars in losses worldwide each year (*3*). Therefore, fracture and fatigue resistance of materials is one of the fundamental material properties of interest for structural design and to manage the integrity of infrastructure such as bridges, pipelines, automobiles, airplanes, ships, power plants, etc. (*4*, *5*), that form the “infrastructural backbone” of society.

A typical measure of resistance to fracture is fracture toughness, which represents the energy required to create new crack surface area at the point of incipient or limited crack growth (*4*). Brittle materials are unable to sustain stable crack growth, while ductile materials (including many structural metals) may be capable of substantial crack growth prior to catastrophic failure (*4*). The toughness of materials emerges from the response of the material microstructure to the extreme stresses in the neighborhood near the tip of a crack or crack tip–microstructure interactions—resulting in damage evolution (i.e., intrinsic toughness) or an alteration to the crack driving force (i.e., extrinsic toughness) (*2*, *5*). In materials science, there is a challenge between achieving higher strength versus higher toughness (*2*). To overcome this challenge, understanding crack tip microstructural interactions is foundational; hence, studies help elucidate the toughening mechanisms operative in a given system, thereby enabling design of novel materials. This knowledge base is also critical for developing physics-based models for managing the integrity and reliability of structures.

Among the various infrastructural engineering materials, steels have a wide range of application in buildings, bridges, pipelines, and power plants; aluminum alloys in automobile engines and airplanes; and super alloys in aeroengines (*1*). These materials systems of interest are hierarchical and microstructurally complex, composed of a metal matrix and hard second phases such as inclusions, carbides, and intermetallic precipitates (*6*–*9*). Therefore, during fracture, material deformation and damage occur at multiple length scales near and ahead of a crack tip, and this crack-affected region is termed as the fracture process zone (FPZ). Furthermore, there continues to be strong interest in studying damage evolution during fracture (*10*, *11*) and the role of hard second phases in the FPZ, as they are recognized to strongly influence damage nucleation and propagation and, hence, fracture toughness in a wide variety of materials such as steels (*12*, *13*), high entropy alloys (*14*), Al alloys (*15*), and composites (*16*). Traditionally, these interactions have been studied using postmortem microscopy studies to infer an understanding of the factors controlling materials performance (*5*, *17*).

In the past decade, x-ray microtomography (XMT) has become a powerful technique to characterize critical features such as porosity, grain structure, damage nucleation, and evolution (*18*). These studies include understanding failure mechanisms in Li-ion batteries (*19*), superconductors (*20*), grain structure and fatigue damage in Ni-based alloys (*21*) and Al alloys (*22*, *23*), and damage in fiber composites (*24*, *25*). Recently, correlative microscopy (CM), such as combining three-dimensional (3D) XMT with microscopy, has emerged as a powerful tool to enhance our understanding of various materials science phenomena, including fracture and fatigue (*26*) and corrosion/pitting in stainless steel (*27*). The majority of the XMT studies have been conducted using dedicated synchrotron x-ray sources with specially built apparatus for supporting CM studies (*18*). While significant advances have been made in laboratory XMT (L-XMT), their use has been predominantly in low-density materials (foams, bones, and rocks) and low-density metals like aluminum. A limited number of studies exist on high-density materials such as steels using L-XMT (*27*–*30*) because of the challenges associated with the strong absorption of x-rays.

**Fig. 1.**
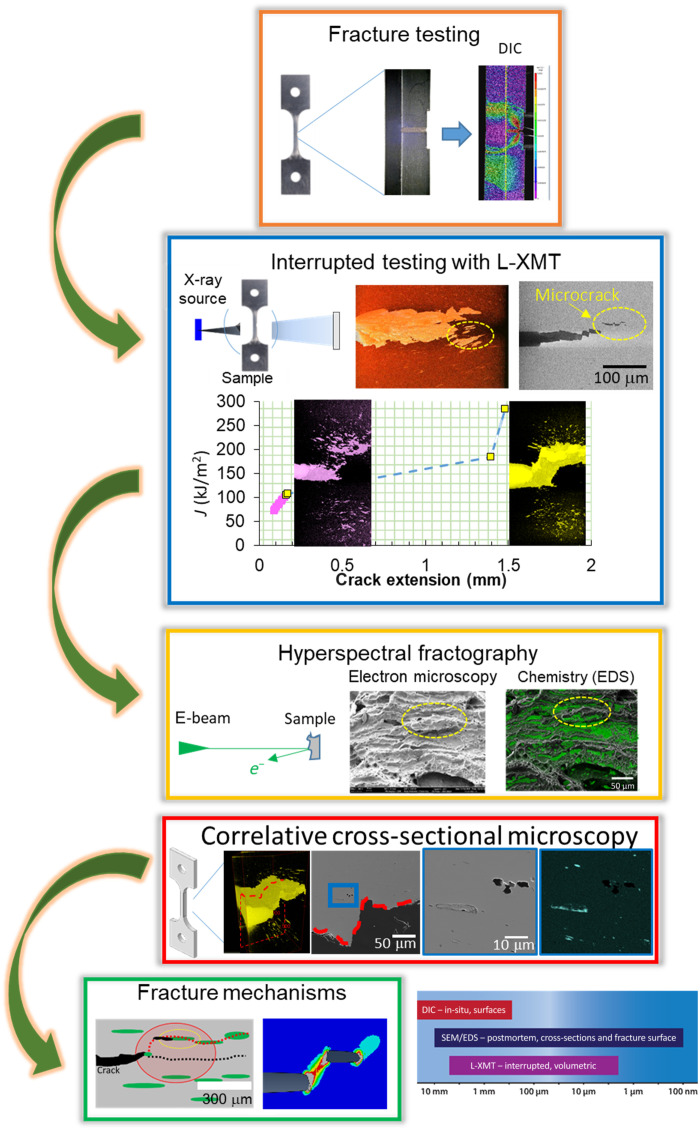
CM workflow. Workflow using L-XMT in combination with interrupted fracture testing used to study damage evolution in the FPZ and fracture mechanisms in steels.

In structural integrity, there is increased recognition that it is important to measure and understand the material fracture resistance in crack tip stress conditions that are representative of service conditions. In the present work, the experiment mimics a surface crack in a pressurized vessel (*28*, *31*, *32*). In this work, we have studied steel inclusion microstructure as a representative system of a ductile matrix with hard second phases to understand crack tip–microstructure interactions in the FPZ during fracture using a single edge notch tension (SENT) specimen. We studied damage evolution from initiation to failure by interrupted testing and characterized the intact sample using L-XMT followed by postmortem CM as depicted in Fig. 1. A summary of the size scales simultaneously assessed by the various correlative techniques used in this work also appears in Fig. 1.

Carbon steel samples were fracture tested using SENT specimens. Digital image correlation (DIC) was conducted during fracture testing to gain insights into fracture processes by monitoring the strain and strain rate field evolution on the specimen surface. Fracture testing with interrupts, allowing for studying incremental crack growth, was conducted, and the sample was then scanned across the full gauge width ahead of a crack nondestructively in L-XMT, with a full field of view of up to 200 mm^3^ at a 2-μm voxel resolution to generate a complete 3D view of the damage state in the FPZ and surrounding material. Hyperspectral microscopy studies were used to validate the L-XMT observations of damage state at a spatial resolution as high as 100 nm and to get additional insights such as the chemistry of inclusions that played a significant role in nucleating and propagating damage in the FPZ. The results from the fracture testing, DIC, L-XMT, and CM studies were combined to gain critical insights into damage evolution in the FPZ and the fracture mechanisms for the model steel inclusion system of the present work. The resulting interpretation of the fracture mechanism was then validated with finite element analyses (FEA). By taking this approach, a more comprehensive assessment of the fracture resistance and its underlying mechanism is obtained. One practical implication of this is the value that it affords engineers by providing greater confidence in their predictions of structural integrity, especially under conditions that may challenge conventional assumptions of such analyses.

The approach developed in this work using L-XMT system with a high resolution and relatively large field of view highlights the possibility of L-XMT–based CM approach to solve materials science challenges that have been limited in the past because of the need to use specialized synchrotron facilities with limited access (*18*, *26*). Furthermore, it can be applied to study progressive damage of materials that can arise from a multitude of degradation phenomena such as creep, oxidation, fracture, and fatigue (or combinations of these). Thus, the ability to use L-XMT for CM studies has the potential for widespread adoption of this approach, thus accelerating research of various materials phenomena, such as fracture.

## RESULTS

**Fig. 2.**
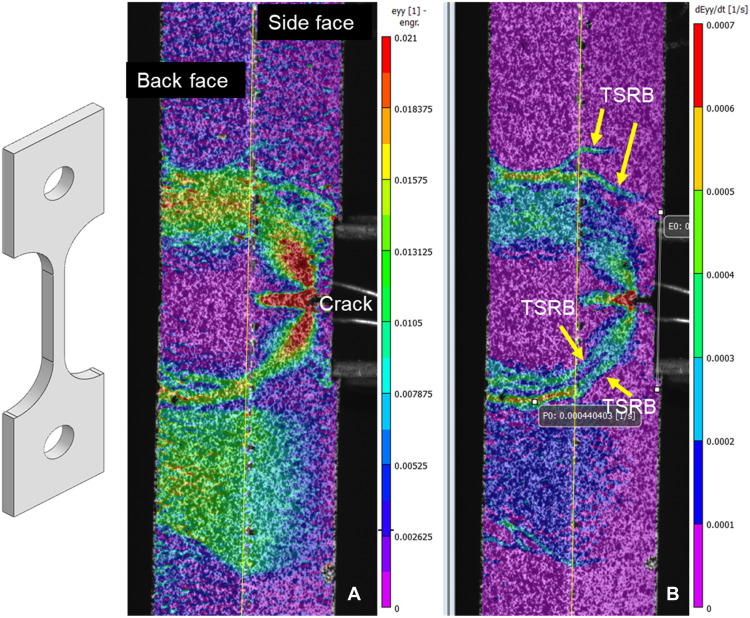
DIC analysis of strain and strain rate during fracture testing of sample A. (**A**) Strain field (in crack opening direction) at peak force obtained by DIC shown in corner-on orientation of sample A, with crack at the right and specimen back face at the left (dotted line denotes corner separating side and back face). (**B**) Strain rate field (in crack opening direction) at peak force. The strain rate field highlights the emergence of TSRBs thought to correspond to underlying microcracking along weak directions in the microstructure. The strain field represents the accumulation of strain rate field up to the present time, blending the contributions of multiple such TSRBs. Face width is 4.7 mm.

DIC mapping of the surface strain and strain rate fields during fracture testing of sample A identified transient strain rate bands (TSRBs) of deformation that emanate from near the crack and extend through the specimen and reach the back face of the specimen (Fig. 2). The strain field shown in Fig. 2A represents the accumulation of both homogeneous deformation and the cumulative contributions of multiple coherent TSRBs such as those in Fig. 2B. These TSRBs, clearly evident in Fig. 2B, are interpreted to indicate the sudden failure of microstructural features in the interior of the sample during fracture testing.

**Fig. 3.**
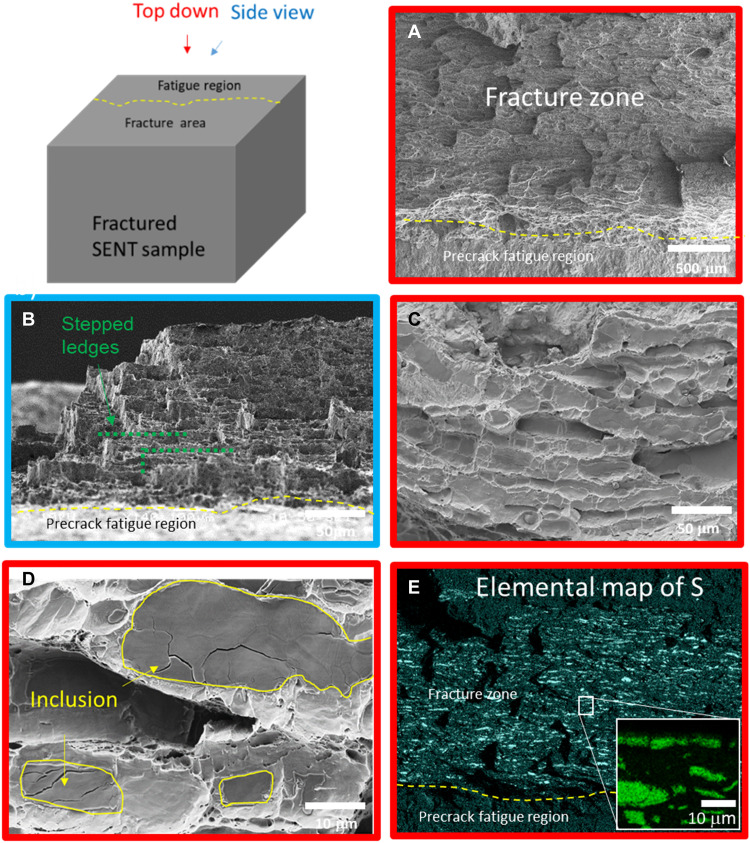
Fractography and microanalysis of fracture surface of sample B. (**A**) Fractograph showing rough/tortuous fracture surface sample B. (**B**) Tilted view relative to top-down view showing many step ledges indicative of the tortuous crack. (**C**) Fractograph showing apparent dimple fracture. (**D**) Higher-magnification view of the dimpled fracture showing the presence of flat inclusions inside the shallow dimples. (**E**) Elemental map of fracture surface shown in (A) with higher magnification of inclusion in inset image confirming the presence of MnS inclusions.

The fracture surface of a companion sample B was initially assessed via conventional posttest fractography, as shown in Fig. 3A. The fracture surface appears rough and tortuous with a generally dimpled surface indicative of ductile fracture. In Fig. 3B, the fracture surface is viewed from a tilted angle, relative to a conventional top-down view to emphasize the unusually high degree of crack path tortuosity. It showed that the fracture surface has a stepped ledge appearance suggestive of failure occurring in planes of weakness in the material. The fracture surface at a higher magnification shows cup and cone features indicative of microvoid coalescence (Fig. 3C). However, it is apparent that the cup and cone features are elongated and also appear to be shallow in depth. Further investigation of the cup and cone features showed what appears to be the presence of flat inclusions inside each dimple or cup feature (Fig. 3D). The inclusions that reside in the elongated cup and cone regions appear to be wide and thin, and some may have cleaved in a brittle manner. An elemental map of the fracture surface showed that a significant area of the fracture surface contained MnS inclusions. The area fraction of MnS inclusions was about 5% of fracture surface compared to an average area fraction of <0.3% of inclusions in the bulk (Fig. 3E).

**Fig. 4.**
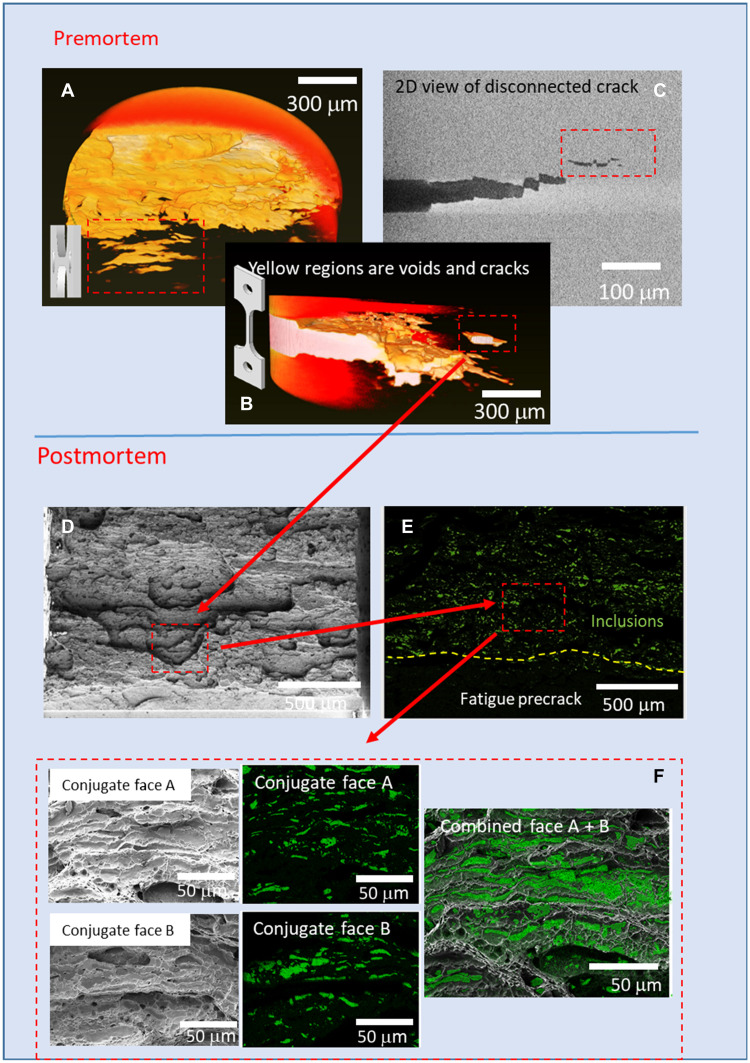
Correlative tomography and fractography of sample C. (**A** and **B**) 3D image of the premortem of sample C revealing distinct microcracks ahead of the main crack tip. (**C**) 2D tomographic slice showing that the disconnected microcrack is coplanar but not in-line with the main crack plane. Note that the correlated regions are indicated by a red dashed bounding box. (**D** and **E**) Following final failure, the postmortem analysis shows that the same microcrack has joined the main crack and is part of overall fracture surface. (**F**) Conjugate fracture surface analysis highlights the rough/tortuous fracture surface, and elemental maps of the conjugate fracture faces were overlaid showing very high projected area fractions of manganese sulfide inclusions including the exact region associated with the microcrack.

To enable a better understanding of the microstructural origin of the TSRBs that were observed during fracture of sample A via DIC studies (see Fig. 2), we conducted an interrupted fracture test in which a fracture sample (sample C) was notched, fatigue precracked, and subjected to a crack driving force sufficient to grow the crack just to incipient tearing and then unloaded. The intact (4.7 mm × 4.7 mm) sample C was imaged through the cross section and across the full crack front of the sample using L-XMT to study the damage state in the FPZ ahead of the crack tip (designated as premortem analysis in Fig. 4, A to C). The sample was then reloaded and pulled to failure.

The 3D image of a subvolume of the intact sample C (premortem) shown in Fig. 4 (A to C) reveals the nucleation of a distinct microcrack ahead of the main crack in the FPZ. In addition, it is apparent that the morphology of the microcrack is irregular and does not show a classic thumbnail geometry. The 2D tomographic cross section in Fig. 4C shows that the microcrack is parallel to but not coplanar with the main crack. It is also not connected to the main crack at this stage but is separated by a ligament of steel. Furthermore, examination of Fig. 4A shows that the microcrack itself is in a state of coalescence (primarily laterally) with other smaller cracks or voids in planes a few micrometers apart parallel to the main crack plane. Following final fracture, postmortem analysis was conducted on the fracture surface, was correlated to premortem tomography data, and is shown in Fig. 4 (D to F). The same microcrack feature shown in Fig. 4 (A to C) could be traced on the final fracture and shown to have coalesced with the main crack and is now part of overall fracture surface (Fig. 4D). Elemental maps of the conjugate fracture faces were overlaid as shown in Fig. 4F to further understand the extent to which the inclusions were present on the final fracture surface. These showed a very high projected area fraction of manganese sulfide inclusions with area fraction of about 35%.

The interrupt experiments showed that microcracks nucleated ahead of the main crack in the FPZ and were inferred to coalesce back to the main crack from postmortem observations. To more directly observe the process of microcrack nucleation and coalescence, we planned a serial interrupt experiment (sample D) with imaging of the damage state through the cross section and across the full crack front of the sample using L-XMT after each interrupt. However, it was noted that there was a significant crack jump almost immediately upon application of the second loading after the first interrupt. This was interpreted as the occurrence of a critical damage event in the FPZ, so the experiment was stopped after the second interrupt, and the damage state was imaged using L-XMT. No further interrupts were conducted, and the sample was fractured in liquid nitrogen (LN_2_) for further CM studies.

**Fig. 5.**
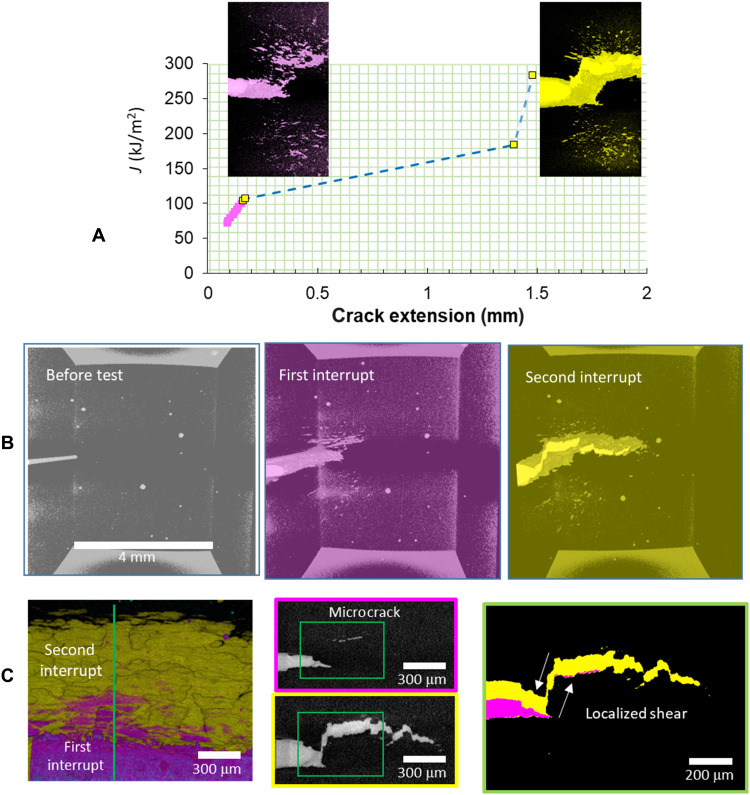
3D tomographic imaging of damage evolution during interrupt testing sample D. (**A**) *J*-*R* curve of an interrupted fracture test (sample D). (**B**) Tomographic volume of the microstructure prior to fracture testing and showing the evolution of the growing main crack and damage ahead of the main crack with the extensive diffuse microcracking in the FPZ. After the second interrupt, one can observe significant crack deflection of the main crack into the diffuse microcrack region. (**C**) The first and second interrupt regions (in pink and yellow, respectively) are overlaid to indicate the extent of the crack growth during each interrupt. The tomographic slices of the crack cross section show the effect of the microcrack in causing the crack deflection. These images are overlaid to show the spatially correlated region of the microcrack and resultant fracture surface, as before the inclusions strongly influence the observed microcracking.

The crack growth resistance curve (*J*-*R* curve) for the serial interrupt test of sample D at the two crack growth positions is shown in Fig. 5A, thus enabling us to capture the fracture driving force associated with the damage states, including the apparent critical damage event that occurred at a fracture driving force of *J* = 107 kJ/m^2^ after the second loading. Figure 5B shows the 3D microstructure prior to fracture testing, indicating the presence of some large and long inclusions away from the main crack. These inclusions serve as fiducial markers for the subsequent interrupt tomographic studies. The damage state and extent of crack propagation after the first (pink) and second (yellow) interrupts are shown in Fig. 5 (B and C). After the first interrupt, we again observed the nucleation of a noncoplanar microcrack in the FPZ similar to that shown in Fig. 4. Upon reloading after the first interrupt, a significant crack jump was observed in the *J*-*R* curve as shown in Fig. 5A. The 3D reconstruction of the crack after the second interrupt shows that the microcrack observed after the first interrupt has coalesced with the main crack (Fig. 5, A and B). Furthermore, a tortuous crack path was observed beyond the location of the initial microcrack (i.e., after interrupt #1), indicating that such a void nucleation–microcrack coalescence process repeated itself during crack growth, albeit on more closely spaced parallel planes. This is clearly apparent in Fig. 5C, where both the first and second interrupt regions are overlaid to indicate the extent of crack growth during each interrupt. The tomographic slices of the cross section of the crack from the same nondestructive dataset show crack deflection via shear localization toward the microcrack and the tortuous crack path beyond the microcrack with apparent stepped ledges. A more comprehensive visual aid to illustrate the extent of damage manifest as the main crack grows from the prefatigue region and the development of diffuse microcracks in tomographic volumes is shown in fig. S1, and the 3D tomographic movies corresponding to Fig. 5 appears in fig. S2.

**Fig. 6.**
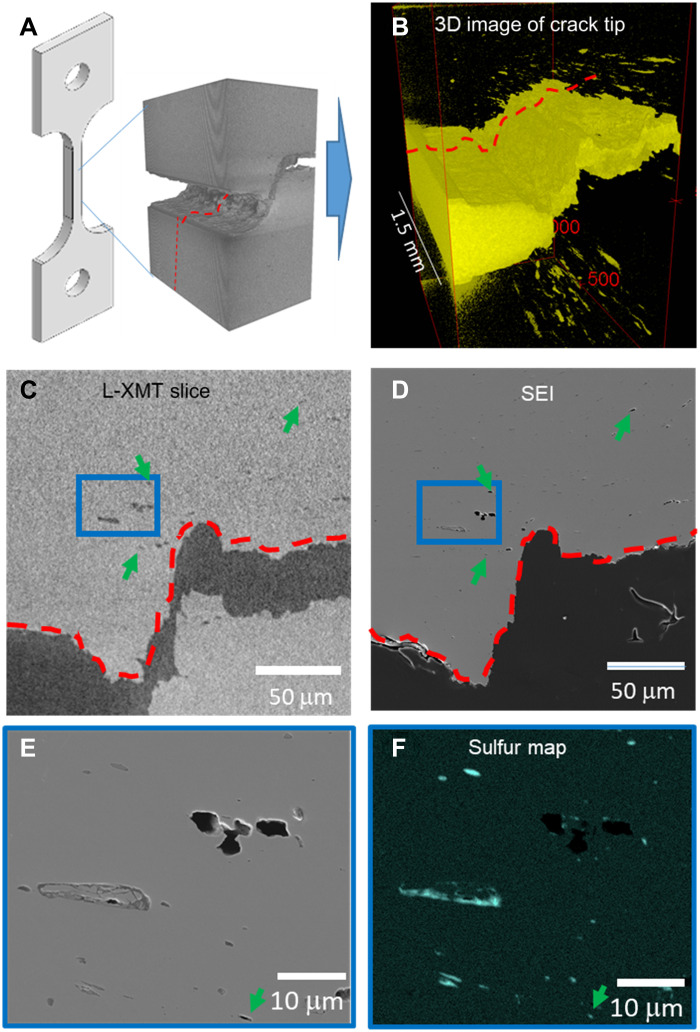
Correlative tomography and cross-section microscopy of sample D. (**A**) Schematic representation of the SENT sample D with the corresponding L-XMT tomographic subvolume. (**B**) 3D tomographic image of the crack tip from the same subvolume showing the correlative cross-sectional slices from the L-XMT (**C**) and SEM images (**D**) indicated by the red dashed line. Many of the larger voids and inclusions observed in SEM were captured in the L-XMT and shown in (C) and (D). (**E**) and (**F**) show a higher-magnification view of the void nucleation and growth around inclusions. Arrows in (C) to (F) point to two examples of the smallest void features that were successfully imaged in the L-XMT.

After the second interrupt, sample D was fractured in LN_2_ and cross-sectioned for CM of the microcracking and damage in the FPZ. Figure 6A shows the orientation of the SENT sample with the corresponding L-XMT tomographic subvolume that was analyzed. The 3D tomographic volume of the crack tip shows the presence of additional void damage in the sample above and below the crack wake after the second interrupt (Fig. 6B). The correlated slices of the crack cross section from L-XMT and scanning electron microscopy (SEM) showing the voiding next to the shear localization region can be seen in Fig. 6 (C and D). The region marked by the blue square in Fig. 6 (C and D) shows the presence of a large inclusion (~16 μm long) that was imaged in the L-XMT volume along with a void cluster next to the inclusion. Higher-magnification SEM image of the inclusion and void cluster and S chemical map is shown in Fig. 6 (E and F, respectively). One can observe that the L-XMT did not resolve the finer inclusions in the volume that can be observed in the SEM slice shown in Fig. 6F and depicted in more detail in fig. S3. However, it can also be observed that some voids as small as 5 to 6 μm were successfully captured in the tomographic slices of the cross section of the crack (three examples are shown by arrows), indicating that fine micrometer-scale voids were captured in the L-XMT volume. Note that the apparent contrast of the larger MnS inclusion in Fig. 6C is similar to a void, whereas the inclusion is visible in the SEM image (Fig. 6E). However, we observed that the MnS inclusions of this size scale are not imaged in the sample ahead of the crack tip before testing (Fig. 5B, before test), indicating that the x-ray attenuation of MnS inclusions is much higher than voids. Furthermore, it is also apparent that the MnS inclusions can be imaged in L-XMT only when voids nucleate and grow around them (Fig. 5B, first and second interrupts). Therefore, the apparent contrast is an artifact because of the MnS inclusion being typically attached to one of side of a larger void around the inclusion and hence producing an average contrast similar to a void.

**Fig. 7.**
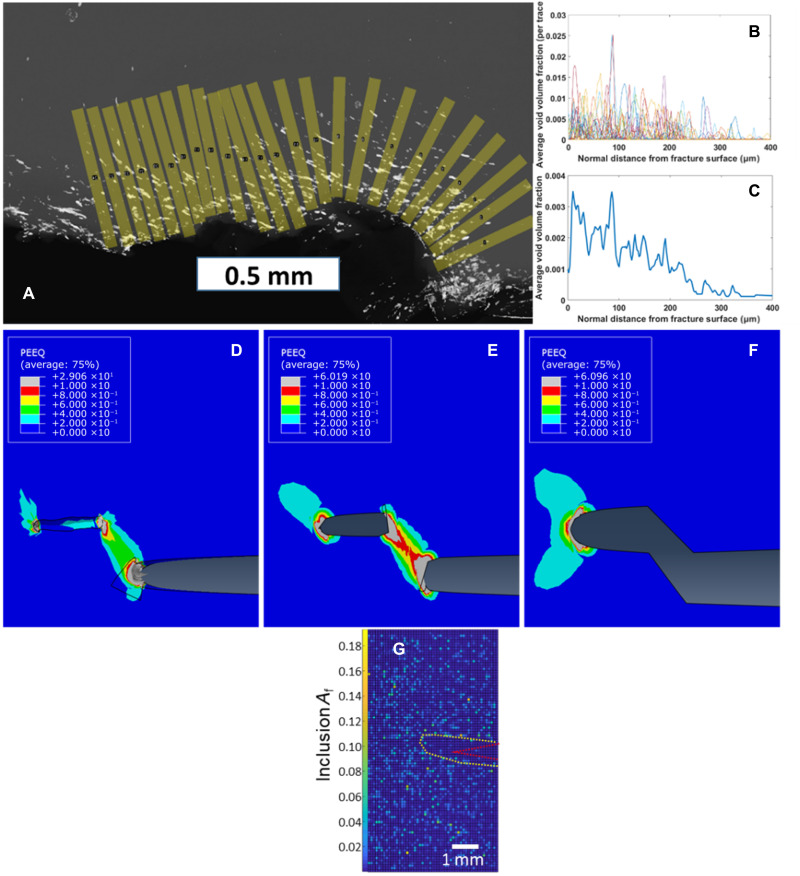
Analysis of damage evolution and fracture behavior in sample D and representative inclusion microstructure of the steel studied. (**A**) Projection of segmented L-XMT data shows the total void density averaged through the specimen thickness after the unstable fracture event in Fig. 5. (**B**) Projected average void volume fraction per trace normal to crack surface shown in (A) shows significant void density peaks corresponding to microcracks. (**C**) Average of void volume fraction from all normal traces in (B) shows the homogenized void population dies out at 300 μm from the fracture surface. (**D** to **F**) FEA results showing effective plastic strain for a 300-μm-long microcrack located 300 μm vertically from the main crack at the predicted moment of failure of the ligament separating the microcrack from the main crack: (D) 3D model with microcrack 800 μm wide; (E) transversely propagated through-thickness microcrack, showing the ligament just exceeding ε_eff_ = 0.8; and (F) connected microcrack and main crack after failure of ligament. The crack driving force ahead of the microcrack scales with the crack tip opening displacement, indicating the rising probability of fracture with progressive failure of material around the microcrack (see text for details). PEEQ, equivalent plastic strain. (**G**) Representative area fraction of MnS inclusions showing the presence of an inclusion-depleted zone (outlined in yellow) at the nominal crack location of *a*/*w* = 0.3 (red). Alternative visualization with areal number density of MnS inclusion is shown in fig. S4.

A postmortem L-XMT scan of specimen D shown in Fig. 6 showed significant elongated void formation in a layer conforming to the fracture surface (Fig. 7A) over a length of about 1.2 mm, which corresponds to the crack advance in the fracture test data in Fig. 5A. The thickness of this void layer is approximately 300 μm, as quantified by the through-thickness–averaged void fraction obtained by machine learning segmentation, mapped as the normal distance from the fracture surface (Fig. 7B). The thickness-averaged void volume fraction shows peaks exceeding 1%, which corresponds to large sheet-like isolated microcracks. Taking the average of all normal traces, the homogenized void fraction peaks at about 0.3% and dies out at about 300 μm from the fracture surface (Figure 7B). The void layer thickness corresponds to the height of the discontinuous jump from the initial crack tip to the tearing surface; this is not unexpected, as the crack driving force and, hence, FPZ size were approximately constant from the moment of tearing onset to the arrest of the crack during tearing and therefore are expected to cause damage over a similar length scale (Fig. 5A). It is also worth commenting that the peak thickness-averaged void volume fraction of 1% is substantially less than the observed areal fraction of inclusions on the fracture surface (Fig. 4F). This is because the averaging scheme integrates over rays in the thickness direction, and the microcracks are stochastically positioned such that a ray that passes through one particular void is otherwise going to primarily pass through steel. The fracture surface is formed by perpendicular jumps between elongated voids containing inclusions, and these jumps have effectively no size when viewed from above. Thus, a fracture surface formed by the progressive coalescence of aligned but noncoplanar elongated voids is expected to have a much greater fraction of inclusions than a 2D or 3D homogenization of the void population would seem to indicate.

FEA simulations were conducted to evaluate the plastic strain and crack driving force in the macro/microcrack system and to clarify the process of discontinuous crack growth in the present material system. FEA results at the predicted main microcrack ligament failure condition (effective plastic strain ε_eff_ > 0.8) are shown for three different crack tip states: the main crack with a microcrack having a width of 0.8 mm and a length of 0.3 mm (Fig. 7D), a laterally expanded “through” microcrack (Fig. 7E), and coalesced main microcrack after ligament failure (Fig. 7F). For this nominal critical condition for ligament failure, the crack driving force of the main crack is *J* = 118 kJ/m^2^ in the absence of a microcrack (such as prior to lateral expansion of the microcrack for most of the specimen), which is in good agreement with the experimental value *J* = 107 kJ/m^2^. After the lateral expansion of the microcrack, the J-integral at the leading edge of the microcrack increases to *J* = 150 kJ/m^2^, and after the joining of the cracks into a single crack, the J-integral at the leading edge of the new crack front increases to *J* = 191 kJ/m^2^. The size of the plastic zone at all crack tips scales with the crack driving force acting there, as does the crack tip opening displacement, to give an additional measure to interpret Figure 7 (D to F). It clearly shows that the driving force at the leading edge of the microcrack prior to lateral expansion is limited. Thus, the failure of the ligament separating the laterally expanded microcrack and main crack instantaneously increases the crack driving force by 62% along the entire crack front. Furthermore, after main crack–microcrack coalescence, the active crack tip is now centered in material with an aligned and higher-density inclusion population, which presumably has a reduced crack growth resistance. Therefore, at the moment of ligament failure, the suddenly increased crack driving force at the leading tip of the microcrack likely substantially exceeds the fracture toughness in that region, resulting in unstable crack growth.

## DISCUSSION

A key observation from this work was the nucleation of microcracks on a plane parallel to and ahead of the main crack plane. We propose that the nucleation of voids around inclusions and their growth into disconnected microcracks are the likely microstructural origin for the observation of TSRBs in the DIC studies. The formation of these microcracks does not appear to be preceded by a strain localization, as observed by Morgeneyer *et al*. (*33*) in Al alloy sheet, although microscopic plastic strain bands could possibly have aided damage nucleation. The sheet-like morphology of damage followed the material microstructure, and not a maximum shear surface, which led to discontinuities in the crack path, in contrast to the more homogeneous failure observed by Morgeneyer *et al.* (*33*). Furthermore, we observed in the fracture test of sample D shown in Fig. 5 that an unstable crack growth occurred upon the second loading. The FEA of the crack/microcrack interaction and the state of local deformation significantly illuminated the phenomenology of fracture in this case. On the basis of the CM and evidence of microcrack formation at subcritical fracture driving forces, we formulate the hypothesis that the failure of the ligament of steel between the main crack and microcrack largely determines the resistance to fracture in the present steel inclusion system. FEA serves to apply a realistic geometry and local failure criterion for this ligament and subsequently evaluate whether this explains the instability observed at 107 kJ/m^2^ (Fig. 5). One effective criterion for ductile local failure is the Hosford-Coulomb failure model, which has been used extensively, including modeling of ductile tearing in fracture experiments, pioneered by Wierzbicki and co-workers (*34*). Experiments on small-scale specimens using a steel similar to that used in the present work, estimated that for a stress triaxiality of approximately *T* ~ 0.8, the effective plastic strain at failure is ε_eff_ ~ 0.8 (*35*). While the strain to failure at stress triaxiality *T* ~ 0.8 is material dependent, the value reported here is consistent with those reported for dual phase (DP) (*36*), HY-100 (*37*), and API X70 (*38*) steels. The stress triaxiality in the region between the main crack and microcrack is between 0.8 and 1. Applying this criterion for the joining of the main crack with the isolated microcrack in the 3D model, the failure of the steel ligament is predicted to occur at a J-integral of *J* = 170 kJ/m^2^ (Fig. 7D), which is significantly higher than the observed experimental value of 107 kJ/m^2^. However, if the microcrack first expands laterally along a microstructural inclusion band through the nucleation of additional microcracks along the crack front and coplanar to the initial microcrack, then the failure of the now much wider steel ligament is predicted to occur at *J* = 117 kJ/m^2^ (Fig. 7E). The crack was observed to grow laterally first as seen from the shape of the microcracks being elongated along the crack front in Figs. 4A, 5C, and 6B, and likely to the width of the specimen during the fracture instability (as evidenced by the fractography shown in Fig. 6C)—this appears to match the interpretation of the FEA results. Failure of the ligament appears to be a necessary condition for fracture, and the FEA results support that it is also a sufficient condition for unstable crack growth, as observed in the experiment. Thus, the failure of the steel ligament separating the microcrack and the main crack is credible as a necessary and sufficient condition for unstable fracture in the steel inclusion system studied in the present work.

The FEA analysis indicated that a critical length scale of a ligament separating the main crack from the microcrack controls the fracture resistance, which is about 300 μm in the steel studied in this work. The width of this ligament is determined by the extent of the inclusion-depleted zone. This is consistent with the microstructure of the steel tested in this work, which showed the presence of an inclusion-depleted zone extending approximately 300 μm from the nominal crack location (Fig. 7G).

The last decade or so has seen a renewed effort in the analysis of the progression of inclusion-related void evolution during failure across multiple prominent metallurgical systems. A review by Antolovich and Armstrong (*3*) covered the conventional approach to ductile damage in 4340, Maraging 200, and HY-100 steels, in which notched tensile specimens with various notch acuities to control the stress triaxiality are examined at various stages of damage with cross-section analysis of void volume fraction and postmortem failure surface analysis (*3*). They also detailed finite element simulations of void-void interactions driving strain localization and go on to promote the notion that a large parametric series of such simulations should be sufficient to virtually assess the ductile damage resistance of any steel inclusion microstructure (*3*). More recent experimental work has taken a renewed look at inclusion-related void growth and coalescence through in situ experiments in notched specimens under synchrotron tomography. In a pair of papers, void growth was tracked in notched tension specimens in a series of aluminum alloys (*15*) and steel alloys (*36*), in which a corrected form of the void growth model of Rice and Tracey (RT) (*39*) was validated against the full population of voids in the specimen, with a voxel size of 1.6 μm leading to a spatial resolution of 4 to 5 μm, assuming that about three voxels are needed to define the smallest feature. While these results were critical for assessing the validity of the RT model in engineering alloys, they were not paired with electron microscopic assessment of damage below the 4-μm scale or accompanying questions of the inclusion population driving void initiation or assisting with void coalescence. This is presumably because the void nucleation and growth occurred from pores and intermetallics in the aluminum alloys, and they could be imaged by tomography (*15*) and from martensite-ferrite interfaces in the steels studied (*36*). A recent paper by Daly *et al*. (*28*) combined high-resolution XMT analyses of void formation in SA508 grade 3 steel with serial sectioning SEM of subvolumes with plasma focused ion beam (FIB) milling. This work extracted 0.5-mm-diameter cores from the fracture surface of a standard compact tension fracture toughness measurement specimen and was able to identify the inclusions as sources of voids both by XMT with a 2- to 3-μm spatial resolution, confirmed by direct observation from the electron microscopy at a resolution of about 75 nm. Furthermore, they identified the population of small carbide inclusions that are thought to assist in ductile damage localization [e.g., Antolovich and Armstrong (*3*)] and confirmed that these are preferentially activated near large inclusion voids. Last, these void populations were quantitatively assessed, both as a function of distance from the fracture surface and from the crack tip, a realistic assessment of how the singular strain field near the crack tip drives a gradient of damage. These measurements were undertaken in part to parameterize the Gurson-Tvergaard-Needleman (*40*) void growth and coalescence model directly from the microscopic results, which the authors note are conventionally inferred from a series of finite element simulations rather from direct observation. While the work of Daly and co-workers (*28*) provides an excellent template for how to combine high-resolution 2D and 3D imaging modalities for damage evolution assessment and model validation, their work is somewhat limited by a small interrogated volume and a lack of fracture driving force data or finite element simulations that would provide a clearer picture of the intensity of strain and stress driving damage as a function of distance from the crack tip. Daly and co-workers (*28*) also highlight the value of high-resolution elemental mapping and diffraction analyses available in electron microscopy to be correlated with the damage evolution process. Last, they comment that such correlative microscopic assessments of ductile damage are needed to challenge the form of the predominant conception of void growth and coalescence, particularly when applied to inhomogeneous or anisotropic distributions of inclusions. These final insights by Daly and co-workers (*28*) are particularly on point for the present work, wherein the fracture process is seen to be dominated by an inhomogeneous (banded) distribution of high–aspect ratio inclusions that are predominantly aligned with the direction of crack growth and thus not amenable to conventional models of ductile damage. Thus, the present work highlights the value of CM to understand the natural history of ductile damage and fracture in nonideal metal-inclusion/hard-phase microstructures. Furthermore, the present work also demonstrates the value of making high-resolution XMT observations over a large field of view (up to 200 mm^3^) that highlighted the formation of isolated microcracks, which could have been missed by making observations over smaller field of view of <1 mm^3^. While finite element simulations are undoubtedly valuable for establishing the phenomenology of void coalescence and strain localization [e.g., Antolovich and Armstrong (*3*)], the complexity of the crack interaction with the inhomogeneous and aligned inclusion population of the present work highlights the value of direct observations of fracture mechanisms without prejudice resulting from expectations derived from existing analytical models.

Capabilities and limitations in the use of L-XMT for CM studies of dense materials like steels merit further discussion. One of the key strengths of synchrotron-based tomography is the ability to acquire tomography data faster, of the order of microseconds to seconds, which is needed for studying materials phenomena that evolve rapidly with time such as phase transformations, grain growth, corrosion reactions, or rapid damage evolution. Tomography scans using L-XMT are typically two orders of magnitude longer in scan times than synchrotron sources (*18*) because of lower accelerating voltages and significantly lower beam flux but with comparable maximum voxel resolution (~2 μm). Therefore, L-XMT is complementary to synchrotron-based XMT and likely well suited to characterize phenomena such as fracture. It is also atypical to tomographically image steel samples with thicknesses of ~5 mm in L-XMT because of the strong attenuation and longer scan times. In the present work, these challenges were overcome with longer exposure times and a dedicated sample alignment fixture to attain the high-spatial resolution while sampling the entire gauge width of the fully intact sample using a local tomography approach, thus using the full capability of the L-XMT systems (see Materials and Methods for details). As discussed earlier, not all inclusions were imaged with tomography, as the steel studied contained inclusions at length scales at and below the resolution of L-XMT (see Fig. 6, D and F, and fig. S3). The nanometer-scale resolution afforded by SEM, transmission EM, and FIB microscopy analysis is thus a necessary complement to XMT and with the maximum resolution needed dictated by the physics of the phenomena being studied. However, in this work, we were able to successfully image voids in the size range of 5 to 6 μm using L-XMT as shown in Fig. 6. We demonstrated the power of CM with L-XMT with a large field of view as it was critical to capture the key phenomenology of microcrack formation, highlighting that fracture resistance was determined by the coalescence of the microcrack with the main crack.

In summary, we have developed a CM framework combining high-resolution hyperspectral SEM with laboratory-based XMT and applied it to the study of fracture mechanisms in a steel inclusion system. We observed damage nucleation and growth to be inhomogeneous and anisotropic. Fracture resistance was observed to be controlled by microstructural factors, such as inclusion distribution and the size scale of an inclusion-depleted zone. Thus, we have again demonstrated that L-XMT systems can characterize damage at the micrometer scale in dense materials such as steels with a large field of view sufficient to scan an entire full-sized fracture specimen with a spatial resolution of 5 to 6 μm. Furthermore, we showed that such CM studies need not be limited to synchrotron facilities. The approach developed in this work can be applied to study progressive damage of materials that can arise from a multitude of different degradation phenomena such as creep, oxidation, fracture, and fatigue. L-XMT has the promise to greatly accelerate discovery and development of new materials and modeling of materials systems, particularly when combined with electron imaging modalities in a CM framework.

## MATERIALS AND METHODS

SEM was performed on an LEO 1530 field emission gun SEM and JEOL tungsten filament. The SEM was operated at 20 kV for standard secondary electron imaging at low magnifications and at 5 kV for imaging at high magnifications and for high-resolution elemental mapping using energy-dispersive spectrometry in an EDAX Octane Elite silicon drift detector (SDD) with a silicon nitride (Si_3_N_4_) window. Imaging and analytical work was also performed on a Zeiss Auriga Crossbeam 540 SEM with a Schottky field emission source and an EDAX Octane Super silicon drift detector, which is a 60-mm^2^ energy-dispersive x-ray spectrometer. For large area imaging, we used the on-board scanning engine of the microscope to produce single images up to 32,768 × 24,576 pixels as well as the NanoPatterning and Visualization Engine software package to produce stitched montages of individual 16,384 × 16,384 pixel fields. The microscope’s on-board depth of field mode was also enabled to maintain focus across the large area. The specific detectors used to acquire these images varied depending on the desired contrast mechanisms but included an Everhart-Thornley detector, solid state backscatter detector, and an in-lens secondary electron detector.

XMT scans were acquired using a Zeiss Versa 510 micro-CT unit, operating at 160 kV for x-ray and voxel sizes in the range of 2 to 4.5 μm depending upon the required spatial resolution and region of interest. This instrument was used for both postmortem XMT scans and the interrupted testing XMT scans. X-ray projection images were recorded with exposure times in the range of 10 to 50 s per image, with typically 1600 images in one slow stepwise rotation of the sample. Reconstruction of 3D volume datasets was performed with manufacturer-supplied software using the Feldkamp-Davis-Kress–filtered back-projection algorithms, using a beam hardening correction factor of 0.5. Avizo software 9.0 was used to construct the tomographic images presented in Results. To assess the entirety of the crack in each sample while retaining a 2-μm voxel resolution, three separate subvolumes (in the form a cylindrical volume ~2 mm in height and 2 mm in diameter) were acquired along the complete length of the crack, which extended across the entire sample width (e.g., 4.7 mm) using a local tomography approach. Each subvolume was overlapped by ~0.5 mm with its neighboring subvolume to ensure that the crack was fully imaged. A routine was developed to align these volumes in all directions. First, a surface rendering was performed followed by a geometric fit of several landmark features. Landmark planes were identified as bottom of the notch, nearest face, and front face; a sequential fit was performed with the notch landmark as the primary feature. The result is a layered view of each interrupt step within the experiment. We were able to visualize the crack progression from a relative fixed surface, i.e., the bottom of the notch.

Postmortem XMT scans were also acquired with a Nikon XT H 225 ST fitted with a transmission target to achieve a voxel size of 2.05 μm. The 2850 × 2850 pixel detector allows for the full specimen thickness to be imaged at once at this magnification. X-ray projection images were recorded using exposure times of 12 to 40 s per image with typically 2000 images. The data were reconstructed with Nikon’s software package CT Pro, and VGStudio Max by Volume Graphics was used to reduce noise and visualize in 3D. Segmentation of the void and microcrack population was conducted with the Trainable Weka Segmentation algorithm (*41*) in Fiji/ImageJ (*42*), and subsequent void quantification was also conducted in ImageJ.

At the beginning of each scan, the following preprocessing steps were implemented as part of the acquisition protocols. The sample was moved out of the field of view, and an exposure check was conducted by the XMT system to ensure that the detector is not saturated when collecting the reference images. This exposure check limited the exposure time when collecting the reference images to avoid saturating the detector. The reference image is an image of the air using the same scanning settings for the tomography scan. During reconstruction, the air signal was subtracted out from the tomography to prevent any air artifacts.

Fracture testing was conducted on a pin-loaded single-edge notched tension specimen 4.7 mm in width and thickness, with the crack propagation direction aligned with the predominant orientation of the microstructural inclusions. The specimen was loaded in an Instron 8800 load frame (Instron, Norwood, MA); the crack growth was measured using direct-current potential drop (DCPD), and the crack driving force was measured using the fracture technology associates (FTA) Nonlinear Fracture test control program (Laboratory Testing Inc., Hatfield, PA) according to the procedure in ASTM E1820 (ASTM 2020) and specimen parameters reported by Cravero and Ruggieri (*31*).

Finite element simulations were conducted to evaluate the plastic strain and crack driving force in the macro/microcrack system using Abaqus v6.16, Dassault Systèmes, Waltham, MA. An incremental, piecewise-defined J_2_ plasticity model was used, which was calibrated to experimental tensile deformation data, and the model was extrapolated beyond the onset of necking to large plastic strains following a fit to the Ramberg-Osgood plastic hardening law (*32*). Both 2D plane strain and 3D simulations were used using quadratic quadrilateral elements with reduced integration. 3D simulations were conducted for the case of a contained microcrack, and 2D plane strain analyses were conducted for through-crack cases. The actual fracture test specimen geometry was modeled, and details of the modeling approach (geometry, boundary conditions, etc.) can be found in a prior publication (*32*). The size and location of the microcrack (as seen in Fig. 5C) were approximated from the microscopy results—the microcrack is 300 μm long, 800 μm wide, and 300 μm vertically and 200 μm horizontally separated from the initial main crack tip. In all cases, crack tips were modeled with degenerate quadratic elements (duplicate crack tip nodes, midpoint node moved to quarter point) to enforce an elastoplastic crack tip stress singularity and realistic crack tip opening. The J-integral was calculated with an integral contour extending to the backside of the specimen and incorporating all crack-induced plasticity to ensure path independence. No damage model was invoked in the analysis, and the equivalent plastic strain and stress therefore reflect the nominal undamaged condition.

DIC was performed with VIC-3D (Correlated Solutions, Irmo, SC) using a stereoscopic camera setup to measure the strain field on the lateral and backside surface of the fracture specimen. High-sensitivity 12 MP Grasshopper USB3 complementary metal-oxide semiconductor cameras were used (FLIR, Wilsonville, OR) with macro-optics and brilliant light-emitting diode lighting to minimize the camera shutter time and image blurring. Paint speckles were applied to the specimen with an airbrush, with an approximate speckle diameter of 60 to 100 μm. The resolution on the surface of the specimen is 11.5 μm per pixel (in the vertical direction). The correlation window was 21 pixels, the step size was two pixels, and the strain was calculated with a 5-point kernel. This gives a virtual strain gage size of 29 pixels, which translates to an approximated spatial resolution for the strain field of 0.33 mm. The strain rate was calculated directly from the strain history at each point without spatial or temporal smoothing beyond that inherent to the time step between image frames.

## References

[1] M. F. Ashby, D. R. H. Jones, *Engineering Materials 1: An Introduction to Properties, Applications and Design* (Elsevier Science, 2012).

[2] R. O. Ritchie, The conflicts between strength and toughness. Nat. Mater. 10, 817–822 (2011). 22020005 10.1038/nmat3115

[3] S. D. Antolovich, R. W. Armstrong, Plastic strain localization in metals: Origins and consequences. Progress Mater. Sci. 59, 1–160 (2014).

[4] R. W. Hertzberg, *Deformation and Fracture Mechanics of Engineering Materials* (John Wiley and Sons, 1996).

[5] S. Suresh, *Fatigue of Materials* (Cambridge Univ. Press, 2012).

[6] G. B. Olson, Computational design of hierarchically structured materials. Science 277, 1237–1242 (1997).

[7] S. G. Lee, B. Kim, W. G. Kim, K. K. Um, S. Lee, Effects of Mo addition on crack tip opening displacement (CTOD) in heat affected zones (HAZs) of high-strength low-alloy (HSLA) steels. Sci. Rep. 9, 229 (2019). 30659277 10.1038/s41598-018-36782-6 PMC6338775

[8] M. Koyama, Z. Zhang, M. Wang, D. Ponge, D. Raabe, K. Tsuzaki, H. Noguchi, C. C. Tasan, Bone-like crack resistance in hierarchical metastable nanolaminate steels. Science 355, 1055–1057 (2017). 28280201 10.1126/science.aal2766

[9] C. H. Caceres, J. R. Griffiths, Damage by the cracking of silicon particles in an Al-7Si-0.4Mg casting alloy. Acta Materialia 44, 25–33 (1996).

[10] B. Albertazzi, N. Ozaki, V. Zhakhovsky, A. Faenov, H. Habara, M. Harmand, N. Hartley, D. Ilnitsky, N. Inogamov, Y. Inubushi, T. Ishikawa, T. Katayama, T. Koyama, M. Koenig, A. Krygier, T. Matsuoka, S. Matsuyama, E. McBride, K. P. Migdal, G. Morard, H. Ohashi, T. Okuchi, T. Pikuz, N. Purevjav, O. Sakata, Y. Sano, T. Sato, T. Sekine, Y. Seto, K. Takahashi, K. Tanaka, Y. Tange, T. Togashi, K. Tono, Y. Umeda, T. Vinci, M. Yabashi, T. Yabuuchi, K. Yamauchi, H. Yumoto, R. Kodama, Dynamic fracture of tantalum under extreme tensile stress. Sci. Adv. 3, e1602705 (2017). 28630909 10.1126/sciadv.1602705 PMC5457031

[11] J. Coakley, A. Higginbotham, D. McGonegle, J. Ilavsky, T. D. Swinburne, J. S. Wark, K. M. Rahman, V. A. Vorontsov, D. Dye, T. J. Lane, S. Boutet, J. Koglin, J. Robinson, D. Milathianaki, Femtosecond quantification of void evolution during rapid material failure. Sci. Adv. 6, eabb4434 (2020). 33328222 10.1126/sciadv.abb4434 PMC7744076

[12] A. Gupta, A. Cecen, S. Goyal, A. K. Singh, S. R. Kalidindi, Structure-property linkages using a data science approach: Application to a non-metallic inclusion/steel composite system. Acta Mater. 91, 239–254 (2015).

[13] A. Srivastava, L. Ponson, S. Osovski, E. Bouchaud, V. Tvergaard, A. Needleman, Effect of inclusion density on ductile fracture toughness and roughness. J. Mech. Phys. Solids 63, 62–79 (2014).

[14] B. Gludovatz, A. Hohenwarter, D. Catoor, E. H. Chang, E. P. George, R. O. Ritchie, A fracture-resistant high-entropy alloy for cryogenic applications. Science 345, 1153–1158 (2014). 25190791 10.1126/science.1254581

[15] E. Maire, S. Zhou, J. Adrien, M. Dimichiel, Damage quantification in aluminium alloys using in situ tensile tests in x-ray tomography. Eng. Fracture Mech. 78, 2679–2690 (2011).

[16] L. J. Huang, L. Geng, H. X. Peng, Microstructurally inhomogeneous composites: Is a homogeneous reinforcement distribution optimal? Prog. Mater. Sci. 71, 93–168 (2015).

[17] D. Hull, *Fractography, Observing, Measuring and Interpreting Fracture Surface Topography* (Cambridge Univ. Press, 1999).

[18] E. Maire, P. J. Withers, Quantitative x-ray tomography. Int. Mater. Rev. 59, 1–43 (2014).

[19] D. P. Finegan, M. Scheel, J. B. Robinson, B. Tjaden, I. Hunt, T. J. Mason, J. Millichamp, M. Di Michiel, G. J. Offer, G. Hinds, D. J. L. Brett, P. R. Shearing, In-operando high-speed tomography of lithium-ion batteries during thermal runaway. Nat. Commun. 6, 6924 (2015). 25919582 10.1038/ncomms7924 PMC4423228

[20] C. Barth, B. Seeber, A. Rack, C. Calzolaio, Y. Zhai, D. Matera, C. Senatore, Quantitative correlation between the void morphology of niobium-tin wires and their irreversible critical current degradation upon mechanical loading. Sci. Rep. 8, 6589 (2018). 29700359 10.1038/s41598-018-24966-z PMC5920112

[21] S. Gustafson, W. Ludwig, P. Shade, D. Naragani, D. Pagan, P. Cook, C. Yildirim, C. Detlefs, M. D. Sangid, Quantifying microscale drivers for fatigue failure via coupled synchrotron x-ray characterization and simulations. Nat. Commun. 11, 3189 (2020). 32581264 10.1038/s41467-020-16894-2 PMC7314802

[22] A. D. Spear, S. F. Li, J. F. Lind, R. M. Suter, A. R. Ingraffea, Three-dimensional characterization of microstructurally small fatigue-crack evolution using quantitative fractography combined with post-mortem x-ray tomography and high-energy x-ray diffraction microscopy. Acta Mater. 76, 413–424 (2014).

[23] D. P. Naragani, P. A. Shade, P. Kenesei, H. Sharma, M. D. Sangid, X-ray characterization of the micromechanical response ahead of a propagating small fatigue crack in a Ni-based superalloy. Acta Mater. 179, 342–359 (2019).

[24] I. Hanhan, R. F. Agyei, X. Xiao, M. D. Sangid, Predicting microstructural void nucleation in discontinuous fiber composites through coupled in-situ x-ray tomography experiments and simulations. Sci. Rep. 10, 3564 (2020). 32107430 10.1038/s41598-020-60368-w PMC7046650

[25] E. V. Iarve, K. Hoos, M. Braginsky, E. Zhou, D. H. Mollenhauer, Progressive failure simulation in laminated composites under fatigue loading by using discrete damage modeling. J. Composite Mater. 51, 2143–2161 (2017).

[26] T. L. Burnett, P. J. Withers, Completing the picture through correlative characterization. Nat. Mater. 18, 1041–1049 (2019). 31209389 10.1038/s41563-019-0402-8

[27] T. L. Burnett, S. A. McDonald, A. Gholinia, R. Geurts, M. Janus, T. Slater, S. J. Haigh, C. Ornek, F. Almuaili, D. L. Engelberg, G. E. Thompson, P. J. Withers, Correlative tomography. Sci. Rep. 4, 4711 (2015). 10.1038/srep04711 PMC3988479 24736640

[28] M. Daly, T. L. Burnett, E. J. Pickering, O. C. G. Tuck, F. Léonard, R. Kelley, P. J. Withers, A. H. Sherry, A multi-scale correlative investigation of ductile fracture. Acta Mater. 130, 56–68 (2017).

[29] N. Limodin, J. Réthoré, J.-Y. Buffière, A. Gravouil, F. Hild, S. Roux, Crack closure and stress intensity factor measurements in nodular graphite cast iron using three-dimensional correlation of laboratory x-ray microtomography images. Acta Mater. 57, 4090–4101 (2009).

[30] N. Pathak, J. Adrien, C. Butcher, E. Maire, M. Worswick, Experimental stress state-dependent void nucleation behavior for advanced high strength steels. Int. J. Mech. Sci. 179, 105661 (2020).

[31] S. Cravero, C. Ruggieri, Estimation procedure of J-resistance curves for SE(T) fracture specimens using unloading compliance. Eng. Fract. Mech. 74, 2735–2757 (2007).

[32] J. Furmanski, P. Sarosi, C. Marzinsky, D. Carpenter, N. Thirumalai, Operations, monitoring, and maintenance; materials and joining, in *2020 13th International Pipeline Conference* (2020), vol. 3.

[33] T. F. Morgeneyer, T. Taillandier-Thomas, L. Helfen, T. Baumbach, I. Sinclair, S. Roux, F. J. A. M. Hild, In situ 3-D observation of early strain localization during failure of thin Al alloy (2198) sheet. Acta Materialia 69, 78–91 (2014).

[34] Y. Bao, T. Wierzbicki, On fracture locus in the equivalent strain and stress triaxiality space. Int. J. Mech. Sci. 46, 81–98 (2004).

[35] M. B. Gorgi, J. Furmanski, D. Mohr, From macro- to micro-experiments: Specimen-size independent identification of plasticity and fracture properties. Int. J. Mechan. Sci. 199, 106389 (2021).

[36] C. Landron, E. Maire, O. Bouaziz, J. Adrien, L. Lecarme, A. Bareggi, Validation of void growth models using x-ray microtomography characterization of damage in dual phase steels. Acta Mater. 59, 7564–7573 (2011).

[37] J. Bandstra, D. Goto, D. Koss, Ductile failure as a result of a void-sheet instability: Experiment and computational modeling. Mater. Sci. Eng. A 249, 46–54 (1998).

[38] M. Paredes, T. Wierzbicki, P. Zelenak, Prediction of crack initiation and propagation in X70 pipeline steels. Eng. Fract. Mech. 168, 92–111 (2016).

[39] J. R. Rice, D. M. Tracey, On the ductile enlargement of voids in triaxial stress fields. J. Mech. Phys. Solids 17, 201–217 (1969).

[40] V. Tvergaard, A. Needleman, Analysis of the cup-cone fracture in a round tensile bar. Acta Metall. 32, 157–169 (1984).

[41] I. Arganda-Carreras, V. Kaynig, C. Rueden, K. W. Eliceiri, J. Schindelin, A. Cardona, H. Sebastian Seung, Trainable Weka Segmentation: A machine learning tool for microscopy pixel classification. Bioinformatics 33, 2424–2426 (2017). 28369169 10.1093/bioinformatics/btx180

[42] J. Schindelin, I. Arganda-Carreras, E. Frise, V. Kaynig, M. Longair, T. Pietzsch, S. Preibisch, C. Rueden, S. Saalfeld, B. Schmid, J.-Y. Tinevez, D. J. White, V. Hartenstein, K. Eliceiri, P. Tomancak, A. Cardona, Fiji: An open-source platform for biological-image analysis. Nat. Methods 9, 676–682 (2012). 22743772 10.1038/nmeth.2019 PMC3855844

