# Supplementary material for: Damage evolution during fracture by correlative microscopy with hyperspectral electron microscopy and laboratory-based microtomography
Source: Sci Adv. 2022 Apr 6;8(14):eabj6738. doi: 10.1126/sciadv.abj6738 (PMC8986107; doi:10.1126/sciadv.abj6738)
Supplement: Supplementary file 1 — Figs. S1 to S4 [file sciadv.abj6738_sm.pdf]

Science Advances
AAAS

Supplementary Materials for

Damage evolution during fracture by correlative microscopy with hyperspectral electron microscopy and laboratory-based microtomography

Peter M. Sarosi, Jevan Furmanski, William C. Reese, Donald L. Carpenter, Mikel A. Nittoli, Michael G. Myers, Nicole M. Callen, Thirumalai Neeraj*

*Corresponding author. Email: neeraj.s.thirumalai@exxonmobil.com

Published 6 April 2022, *Sci. Adv.* **8**, eabj6738 (2022)
DOI: 10.1126/sciadv.abj6738

The PDF file includes:

Figs. S1 to S4
Legends for movies S1 to S3

Other Supplementary Material for this manuscript includes the following:

Movies S1 to S3

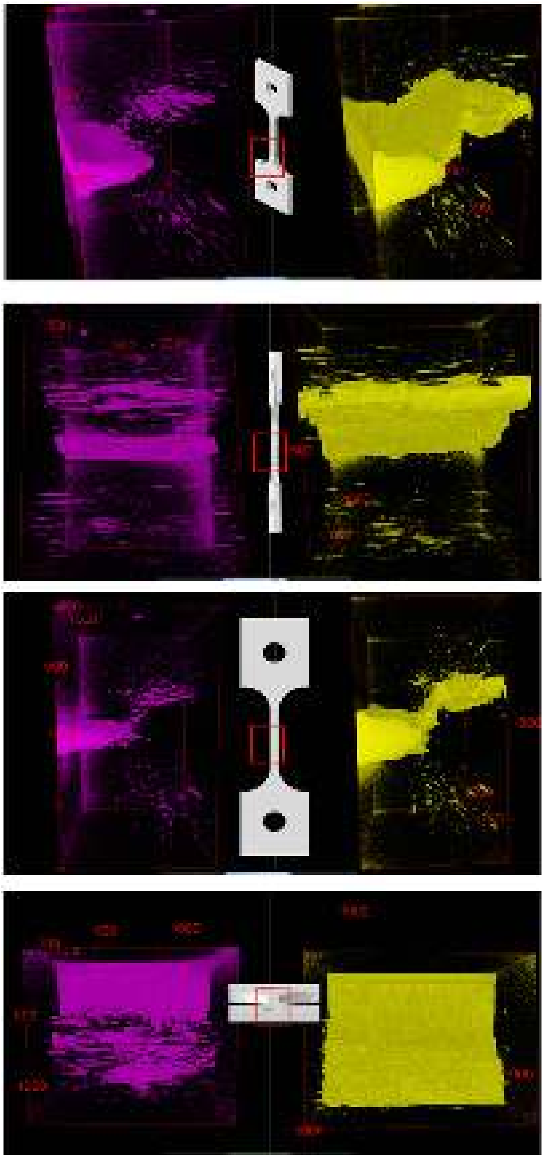

Fig. S1. Additional views of damage evolution during interrupt testing of sample D shown in Figure 5. 3D views of fracture process zone (FPZ) ahead of the main crack in different orientations after interrupt testing in sample D. The first interrupt (left/pink) shows extensive micro-cracking in the FPZ at planes parallel to the main crack plane and broad lateral growth of the cracks prior to forward crack growth. In the second interrupt (right/yellow) one can observe many of micro-cracks have coalesced in plane and also have joined the main crack.

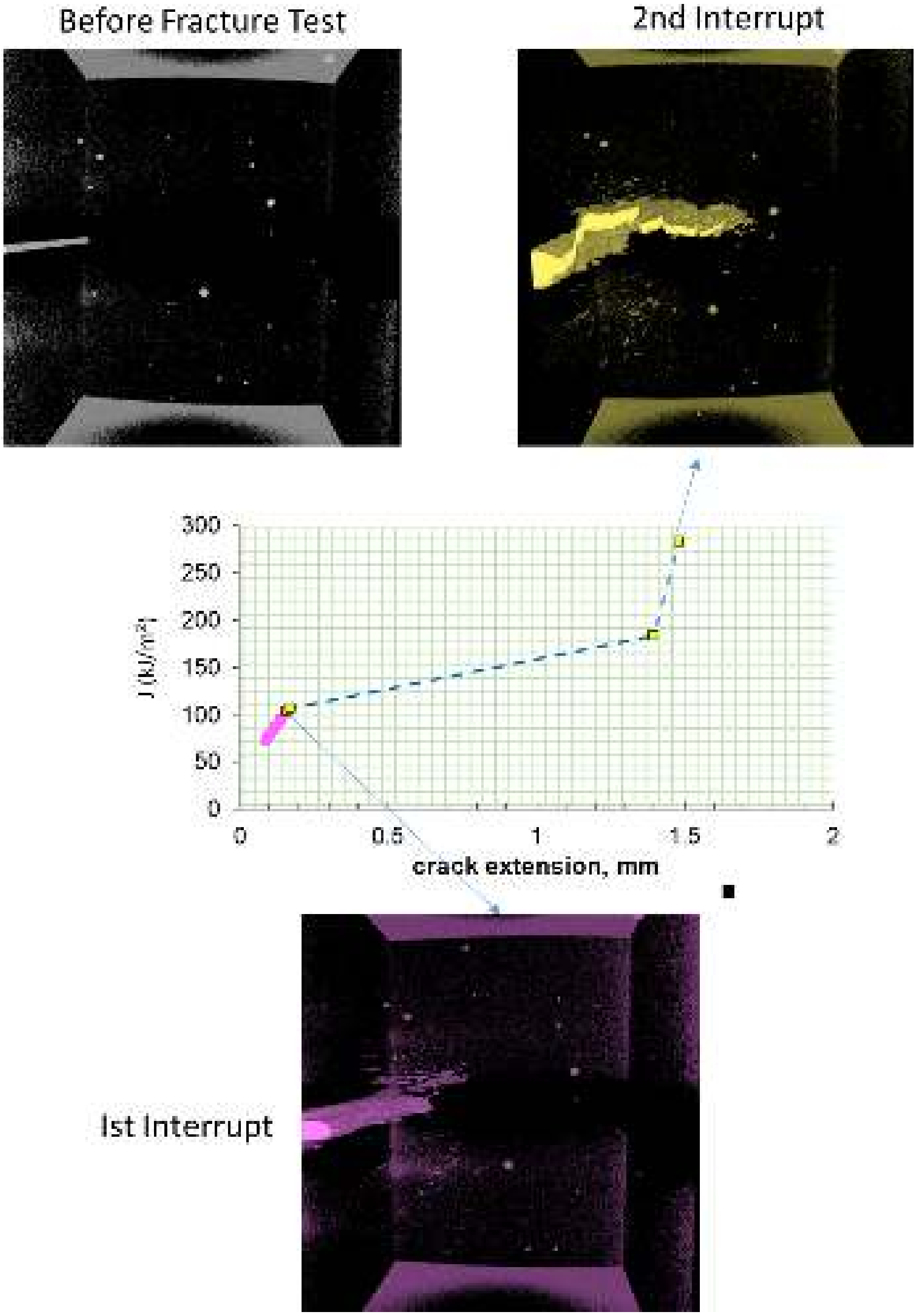

Fig. S2. Movies of damage evolution at different stages of interrupt testing of sample D discussed in Figure 5. 3D Movies of the tomographic scans showing the evolution of damage after two interrupts along the trajectory of the J-R curve of sample D. Bounding box in movie is 5mm.

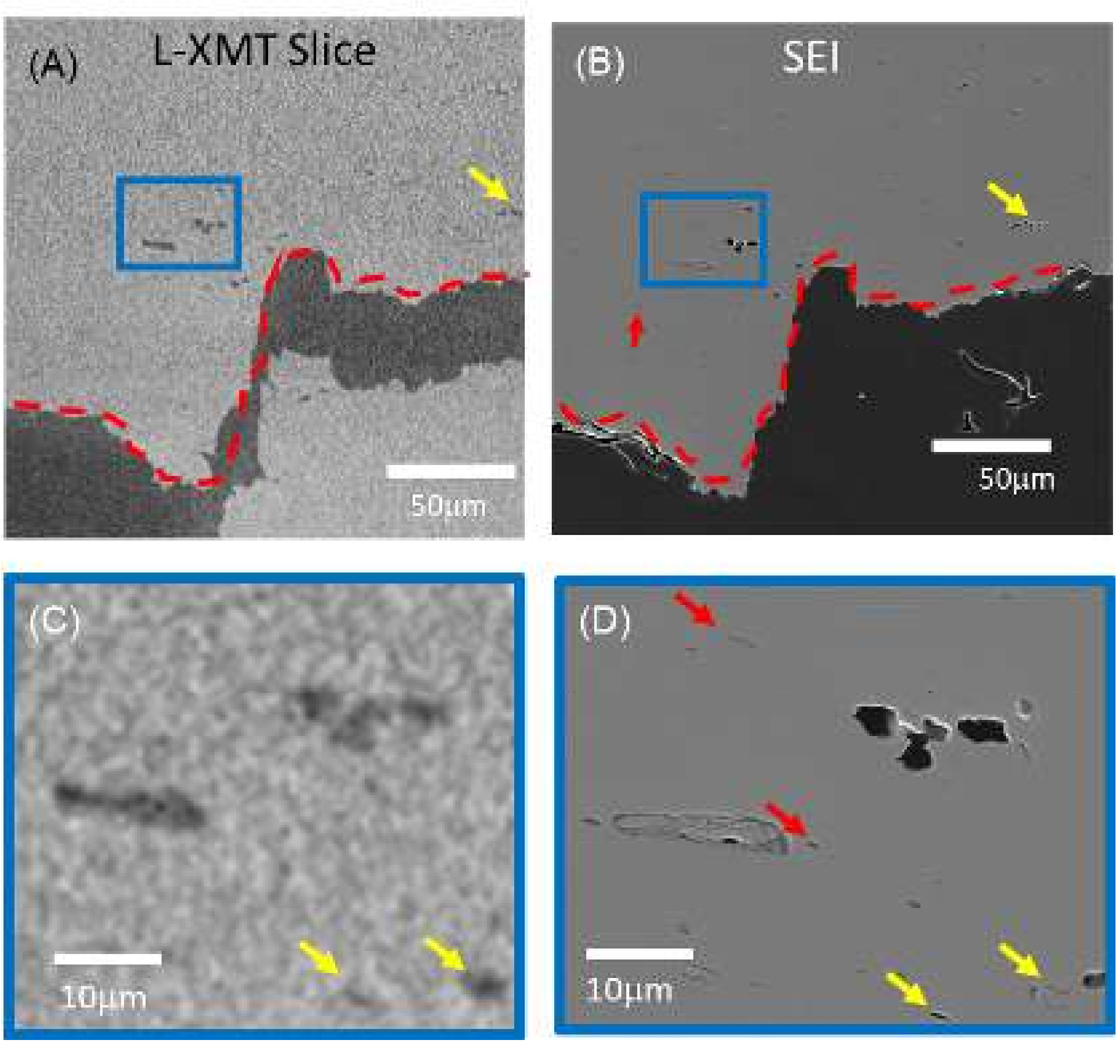

Fig. S3. Correlative tomography and cross-section microscopy of sample D discussed in figure 6. (A)-(D) Images showing the spatial resolution limits of L-XMT compared to SE imaging from sample D. Yellow arrows indicate examples of features observed via both techniques. Red arrows indicate examples of features observable in electron microscopy but not in L-XMT.

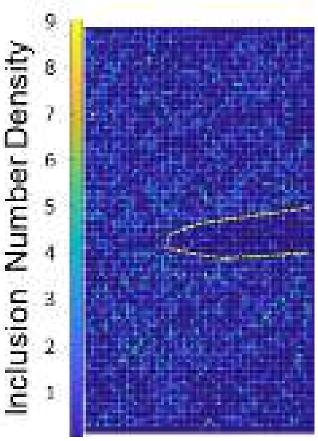

Fig. S4. Alternative visualization of Figure 7G with areal number density of MnS inclusions. Representative areal number density of MnS inclusions showing the presence of an inclusion depleted zone (outlined in yellow) as an alternative visualization to figure 7g.

Movie S1. Tomography movie before testing (gray) of sample D (see Figure 5 and text for details).

.

Movie S2. Tomography movie after first interrupt of J test (pink) of sample D (see Figure 5 and text for details).

Movie S3. Tomography movie after second interrupt of J test (yellow) of sample D (see Figure 5 and text for details).
